# An Essential Role for the Transcription Factor Runx1 in T Cell Maturation

**DOI:** 10.1038/srep23533

**Published:** 2016-03-29

**Authors:** Fan-Chi Hsu, Michael J. Shapiro, Barsha Dash, Chien-Chang Chen, Megan M. Constans, Ji Young Chung, Sinibaldo R. Romero Arocha, Paul J. Belmonte, Meibo W. Chen, Douglas C. McWilliams, Virginia Smith Shapiro

**Affiliations:** 1 Department of Immunology, Mayo Clinic, Rochester, MN, United States

## Abstract

The transcription factor Runx1 has essential roles throughout hematopoiesis. Here, we demonstrate that Runx1 is critical for T cell maturation. Peripheral naïve CD4^+^ T cells from CD4-cre Runx1 cKO mice are phenotypically and functionally immature as shown by decreased production of TNF-α upon TCR stimulation. The loss of peripheral CD4^+^ T cells in CD4-cre Runx1 cKO mice is not due to defects in homeostasis or decreased expression of IL-7Rα, as transgenic expression of IL-7Rα does not rescue the loss of CD4^+^ T cells. Rather, immature Runx1-deficient CD4^+^ T cells are eliminated in the periphery by the activation and fixation of the classical complement pathway. In the thymus, there is a severe block in all aspects of intrathymic T cell maturation, although both positive and negative selection are unaltered. Thus, loss of Runx1 leads to the earliest characterized block in post-positive selection intrathymic maturation of CD4 T cells.

The generation of functional mature T cells relies on post-positive selection T cell maturation^1^. Intra-thymic maturation occurs at the SP stage while post-thymic maturation requires contact with secondary lymphoid organs such as spleen or lymph nodes before T cells enter the long-lived naïve T cell pool^2^. Single-positive (SP) thymocytes can be divided into three populations based on their maturation status: semi-mature (SM) SP thymocytes are CD24^+^ CD69^+^ MHCI^−^CCR7^−^ and are susceptible to death receptor signaling-mediated apoptosis; mature 1 (M1) SP thymocytes are CD24^+^ CD69^+^ MHCI^+^ CCR7^+^ and are resistant to death receptor induced apoptosis and are able to proliferate after TCR stimulation; mature 2 (M2) SP thymocytes are CD24^−^CD69^−^MHCI^+^ CCR7^+^ and gain the ability to egress from the thymus^3^,^4^,^5^. Once T cells egress from the thymus, the youngest T cells in the periphery are termed recent thymic emigrants (RTEs). RTEs continue to under go post-thymic maturation, increasing their ability to produce cytokines upon stimulation, for two to three weeks before entering the long-lived naïve T cell pool^1^. During maturation, T cells also gain resistance to complement-mediated elimination^6^,^7^. Although the signals and molecular mechanisms that regulate T cell maturation are not well understood, recent studies have identified genes that are specifically required for post-positive selection T cell maturation^8^,^9^,^10^,^11^. In particular, mice with a conditional deletion of the transcriptional regulators NKAP (NF-κB activating protein) or HDAC3 (histone deacetylase 3) have a block in T cell maturation^6^,^7^, leading their elimination by complement in the periphery as RTEs. Concurrent with maturation, T cells increase incorporation of sialic acid, in particular α2,8-linked sialic acid, into cell surface glycans. Loss of sialylation, such as experimentally through neuraminidase, leads to binding of natural IgM and activation of complement^12^,^13^. RTEs from CD4-cre NKAP cKO or CD4-cre HDAC3 cKO mice have a defect in α2,8-sialylation as well as decreased expression of the complement regulatory protein CD55 that contribute to their complement-mediated elimination. Altered α2,8-sialylation in the absence of NKAP or HDAC3 in RTEs is due to decreased mRNA expression of sialic acid transferases belonging to the ST8Sia family, in particular ST8Sia6^6^,^7^.

The transcription factor Runx1 (also called AML1/CBFA2/PEBP2αB) belongs to the Runx family of transcription factors that share a highly conserved DNA binding *runx* domain^14^. Runx proteins are associated with the non-DNA-binding cofactor CBFβ that allows stable binding of Runx proteins to target DNA sequences. By binding to the regulatory elements of *Cd4*, *Foxp3* and *Gata3*, Runx1 has multiple roles in T cell development^15^,^16^, generation and suppressive function of regulatory T cells^17^,^18^,^19^,^20^, and Th2 differentiation^21^, respectively. CD4-cre mediated Runx1 deletion leads to a decreased number and percentage of CD4 SP thymocytes, especially in the TCRβ^hi^CD24^lo^ population, and few peripheral CD4^+^ T cells^15^. CD8^+^ T cells are unaffected in CD4-cre Runx1 cKO mice, due to expression of Runx3^16^,^22^. Expression of IL-7Rα is lower in TCRβ^hi^CD69^+^ CD4 SP thymocytes and peripheral Foxp3^−^CD4^+^ conventional T cells^15^. Since IL-7Rα signaling is required for naïve T cell homeostasis and survival, it was thought that Runx1 regulates survival and homeostasis of peripheral CD4^+^ T cells through regulation of IL-7Rα. However, IL-7Rα transgene expression did not rescue peripheral CD4+ T cell numbers in Runx1 cKO mice, although increased proportions of mature CD4 SP thymocytes was reported^15^. Thus, factors other than IL-7 must also have a role.

Here we demonstrate that the primary defect in peripheral T cells in CD4-cre Runx1 cKO mice is in T cell maturation. Although Runx1-deficient CD4 SP thymocytes and CD4^+^ T cells have decreased expression of IL-7Rα, an IL-7Rα transgene cannot rescue the survival or cellularity of CD4^+^ T cells in CD4-cre Runx1 cKO mice. Conventional CD4 SP thymocytes cannot undergo maturation in the absence of Runx1, although positive selection proceeds normally and there is no evidence for increased negative selection. Peripheral Runx1-deficient CD4^+^ T cells are phenotypically and functionally immature, with a severe defect in the ability to produce TNFα upon stimulation. Runx1-deficient CD4^+^ T cells bind IgM, C1q, C4 and C3 and are thus eliminated by complement in the periphery. Therefore, neither alterations in T cell homeostasis, positive selection or negative selection are responsible for the CD4^+^ T cell defect in CD4-cre Runx1 cKO mice, but the defect is due to a severe block in T cell maturation.

## Results

### Defect in peripheral CD4 T cell cellularity in CD4-cre Runx1 cKO mice is not rescued by an IL-7Rα transgene

**Figure 1:**
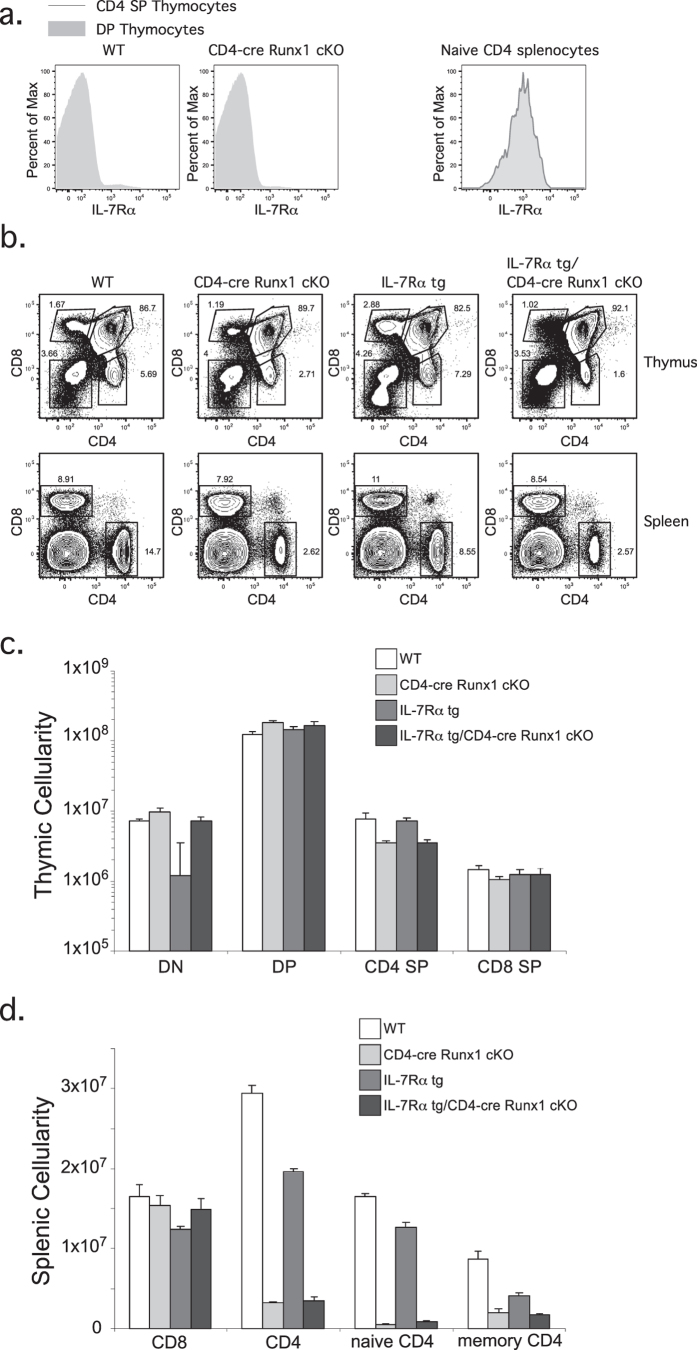
The decrease in peripheral CD4^+^ T cells numbers in CD4-cre Runx1 cKO mice is not due to IL-7Rα mediated homeostasis. (**a**) DP or CD4 SP thymocytes (left) and naïve CD4^+^ splenocytes (right) from WT or CD4-cre Runx1 cKO mice were examined for IL-7Rα expression. DP thymocytes are defined as CD4^+^ CD8^+^; CD4 SP thymocytes are defined as CD4^+^ CD8^−^; naïve CD4^+^ splenocytes are defined as CD4^+^ CD8^−^CD62L^+^ CD44^−^. Representative histograms are shown from five WT and six CD4-cre Runx1 cKO mice from five independent experiments. (**b**) Thymocytes and splenocytes from WT, CD4-cre Runx1 cKO, IL-7Rα tg, and IL-7Rα tg/CD4-cre Runx1 cKO were stained with CD4 and CD8 to examine T cell development. Data is representative from at least four mice in each group from five independent experiments. (**c**) Absolute numbers of DN, DP, CD4 SP, CD8 SP thymocytes, and (**d**) splenic CD4^+^ and CD8^+^ T cells are shown. Quantitation were generated from five WT, six CD4-cre Runx1 cKO, four IL-7Rα tg, and nine IL-7Rα tg/CD4-cre Runx1 cKO mice from five independent experiments. Error bars represent SEM.

Conditional deletion of Runx1 using CD4-cre leads to decreased numbers of CD4 SP thymocytes and peripheral CD4^+^ T cells. The cause of decreased cellularity was initially thought to be due to a defect in T cell homeostasis^15^. Consistent with previous observations, IL-7Rα expression in CD4 SP thymocytes is significantly decreased in the absence of Runx1 (Fig. 1a, left). In the periphery, IL-7Rα expression is decreased by approximately two-fold in Runx1-deficient naïve CD4^+^ T cells compared to WT cells (Fig. 1a, right). If the decreased number of CD4^+^ T cells in CD4-cre Runx1 cKO mice is due to IL-7-mediated T cell homeostasis, it should be rescued at least in part by expression of an IL-7Rα transgene. However, we find that the percentage and cellularity of CD4 SP thymocytes and peripheral CD4^+^ T cells between CD4-cre Runx1 cKO and IL-7Rα tg/CD4-cre Runx1 cKO mice are similar (Fig. 1b–d). In contrast to earlier work^15^, an IL-7Rα transgene does not rescue the development of thymic CD4^+^ T cells. These data suggest that the defect in CD4 lineage T cells in CD4-cre Runx1 cKO mice is due to a mechanism other than IL-7Rα mediated homeostasis.

### Runx1 is required for phenotypic and functional maturation of CD4^+^ T cells

**Figure 2:**
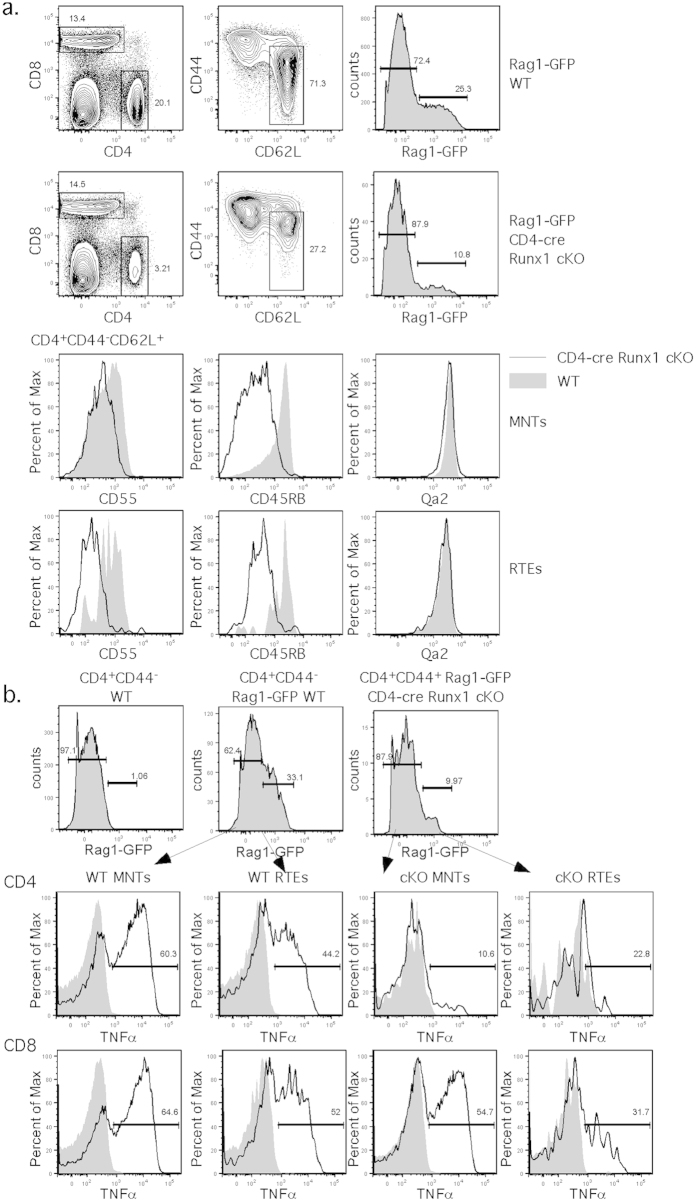
Peripheral CD4^+^ RTEs and MNTs from CD4-cre Runx1 cKO mice are phenotypically and functionally immature. (**a**) CD4^+^ RTEs or MNTs from Rag1-GFP WT or Rag1-GFP CD4-cre Runx1 cKO mice were examined for the expression of the maturation markers, CD55, CD45RB and Qa2. CD4^+^ RTEs were gated on CD62L^+^ CD44^−^Rag1-GFP^+^ while CD4^+^ MNTs were gated on CD62L^+^ CD44^−^Rag1-GFP^−^. Data are representative of seven Rag1-GFP WT and eight Rag1-GFP CD4-cre Runx1 cKO mice from five independent experiments. (**b**) Splenocytes from Rag1-GFP WT or Rag1-GFP CD4-cre Runx1 cKO were either left unstimulated or stimulated overnight with α-CD3ε and α-CD28. The following day, CD44^lo^ CD4 and CD8 Rag1-GFP^−^ MNTs and Rag1-GFP^+^ RTEs were examined for intracellular TNF-α production by flow cytometry. The top row shows the gating strategy for RTEs and MNTs within the naïve CD4 T cell pool. For examination of TNF-α production, the filled histogram represents unstimulated cells, and the solid line represents cells that were stimulated with α-CD3ε/α-CD28. The gate indicates the percentage in each stimulated sample that expresses TNF-α. Data are representative of four Rag1-GFP WT and four Rag1-GFP CD4-cre Runx1 cKO mice from three independent experiments.

Recent thymic emigrants (RTEs) upregulate IL-7Rα expression as they progress to mature native T cells (MNTs) during T cell maturaton^1^,^3^. Mice with defects in T cell maturation have lower levels of IL-7Rα expression^6^,^7^. Thus, the compromised IL-7Rα expression in CD4-cre Runx1 cKO mice may reflect a block in maturation rather than a defect in homeostasis. In order to study T cell maturation in CD4-cre Runx1 cKO mice, we crossed these mice with Rag1-GFP knock-in mice^23^. While RAG1 transcription terminates after positive selection, the long half-life of GFP marks newly egressed RTEs with GFP^1^,^5^,^8^. However, the percentage of CD4^+^ RTEs (CD4^+^ CD62L^+^ CD44^−^Rag1-GFP^+^) is not enriched in CD4-cre Runx1 cKO, and is even less as compared to WT mice (Fig. 2a, top). As RTEs mature, they upregulate CD55, CD45RB and Qa2^1^,^7^. Runx1-deficient CD4^+^ RTEs and MNTs (CD4^+^ CD62L^+^ CD44^−^Rag1-GFP^−^) express lower levels of CD55 and CD45RB, suggesting that Runx1-deficient naïve CD4^+^ T cells have a defect in T cell maturation. Although Qa2 levels were normal, we previously found normal Qa2 expression in CD4-cre HDAC3 cKO mice with a T cell maturation defect^6^, demonstrating that Qa2 expression alone cannot be used as a measure of maturation. We examined the protein expression level of Runx1 throughout T cell development and maturation. CD4 SP thymocytes, and naive and memory CD4^+^ T cells from CD4-cre Runx1 cKO mice have low levels of Runx1 protein expression, indicating a high efficiency of cre-mediated Runx1 deletion. In particular, the peripheral CD4^+^ T cells present in these mice have low levels of Runx1 expression and T cells that escaped cre-mediated deletion were not preferentially present or expanded (Supplemental Fig. 1).

As T cells mature in the periphery, they gain functional competency to produce cytokines such as TNF-α, referred to as licensing^24^,^25^. To determine whether Runx1 is required for functional maturation for T cells, we examined TNF-α production by RTEs and MNTs from Rag1-GFP WT and Rag1-GFP CD4-cre Runx1 cKO mice. Although GFP fluorescence was diminished after fixation and permeabilization, a Rag1-GFP^+^ population could be distinguished from Rag1-GFP^−^ cells using WT mice to set the gate (Fig. 2b). Runx1-deficient CD4^+^ RTEs and CD4^+^ MNTs produce almost no TNF-α upon stimulation, as compared to WT CD4^+^ RTE and CD4^+^ MNTs, indicating that Runx1 is absolutely required for functional maturation of CD4^+^ T cells. Peripheral CD8^+^ T cells from CD4-cre Runx1 cKO mice produce similar amounts of TNF-α as WT CD8^+^ T cells, suggesting a dispensable role of Runx1 in CD8^+^ T cell maturation, likely due to expression of Runx3, which is a master regulator of CD8 lineage differentiation^16^,^22^.

### Peripheral CD4^+^ T cells in CD4-cre Runx1 cKO mice are eliminated by complement

**Figure 3:**
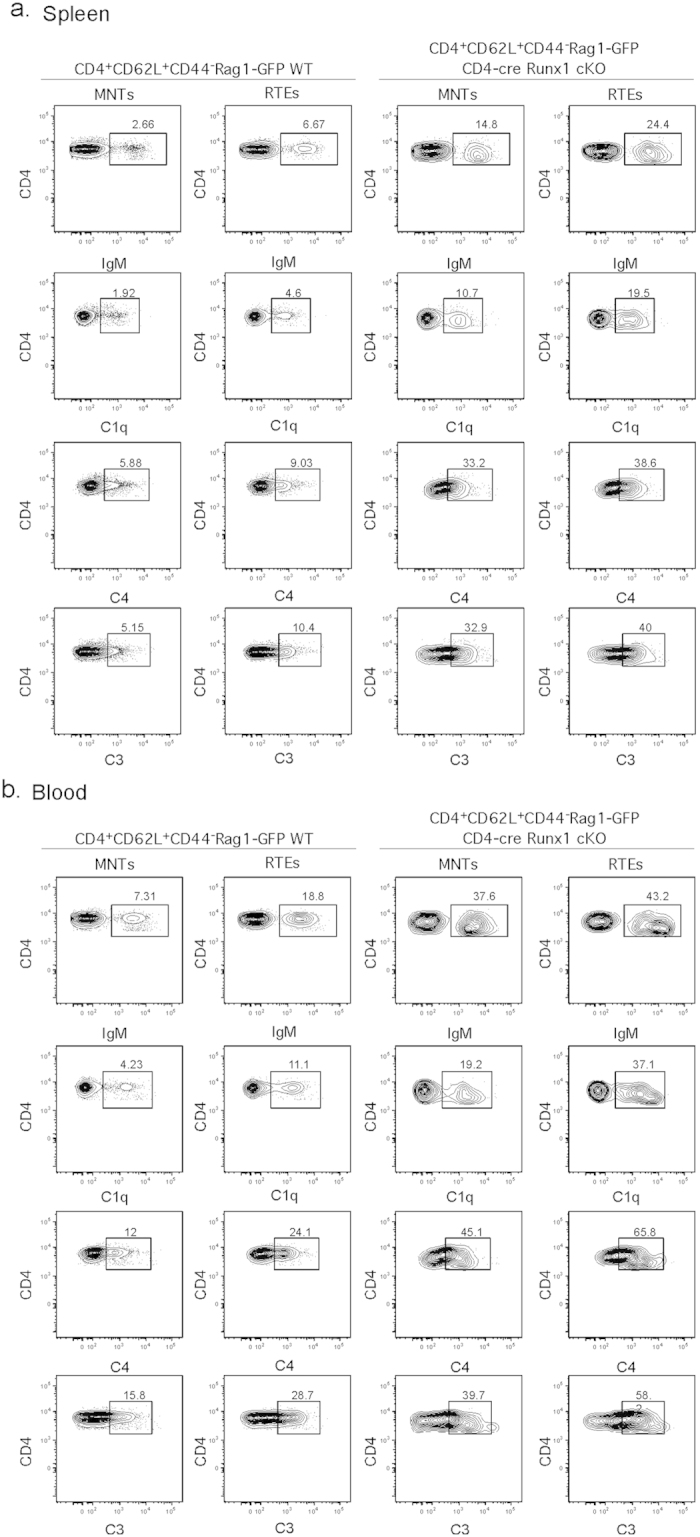
Runx1-deficient peripheral CD4^+^ T cells are targeted by the classical complement pathway. (**a**) Splenic and (**b**) blood CD4^+^ RTEs and MNTs from Rag1-GFP WT and Rag1-GFP CD4-cre Runx1 cKO mice were examined for the deposition of IgM, C1q, C4 and C3 as denoted in the figure. Cells were incubated in the GVB^++^ buffer prior to staining with indicated Abs. CD4^+^ RTEs and MNTs were gated as described in Fig. 2a. Data shown are representative of six Rag1-GFP WT and six Rag1-GFP CD4-cre Runx1 cKO mice from five separate experiments.

We have previously shown that peripheral T cells that fail to mature are eliminated by complement^6^,^7^. Immature T cells from CD4-cre NKAP cKO and CD4-cre HDAC3 cKO mice are cleared by the classical complement pathway, as demonstrated by increased binding of IgM, C1q, C4 and C3. Since CD4^+^ T cells from CD4-cre Runx1 cKO mice are functionally and phenotypically immature, we examined peripheral splenic T cells from Rag1-GFP WT and Rag1-GFP CD4-cre Runx1 cKO mice for IgM binding and complement deposition. Splenic CD4^+^ RTEs and MNTs from Rag1-GFP CD4-cre Runx1 cKO mice have dramatically increased IgM binding and complement C1q, C3 and C4 deposition as compared to CD4^+^ T cells from Rag1-GFP WT mice (Fig. 3a, quantified in Supplemental Fig. 2). Thus, the loss of peripheral CD4^+^ T cells in CD4-cre Runx1 cKO mice is caused by complement-mediated elimination. To determine when complement deposition is initiated, we examined circulating CD4^+^ T cells in blood. Interestingly, we found that Runx1-deficient CD4^+^ RTEs and MNTs from blood have more IgM binding and complement protein deposition than found in the spleen (Fig. 3b compared to Fig. 3a). A significant proportion of WT CD4^+^ RTEs from blood also have increased IgM and complement protein depositions compared to WT splenic CD4^+^ T cells (Supplemental Fig. 2). This suggests that when SP thymocytes egress via the cortico-medullary junction into the circulation^26^, complement initiates elimination of immature T cells from WT mice. Consistent with normal CD8^+^ T cell development and maturation in CD4-cre Runx1 cKO mice, there was no increased IgM binding and complement deposition found on the surface of splenic RTE CD8^+^ T cells from CD4-cre Runx1 cKO mice as compared to WT RTEs (Supplemental Fig. 3).

### CD4 SP thymocytes from CD4-cre Runx1 cKO have a block in intra-thymic maturation

**Figure 4:**
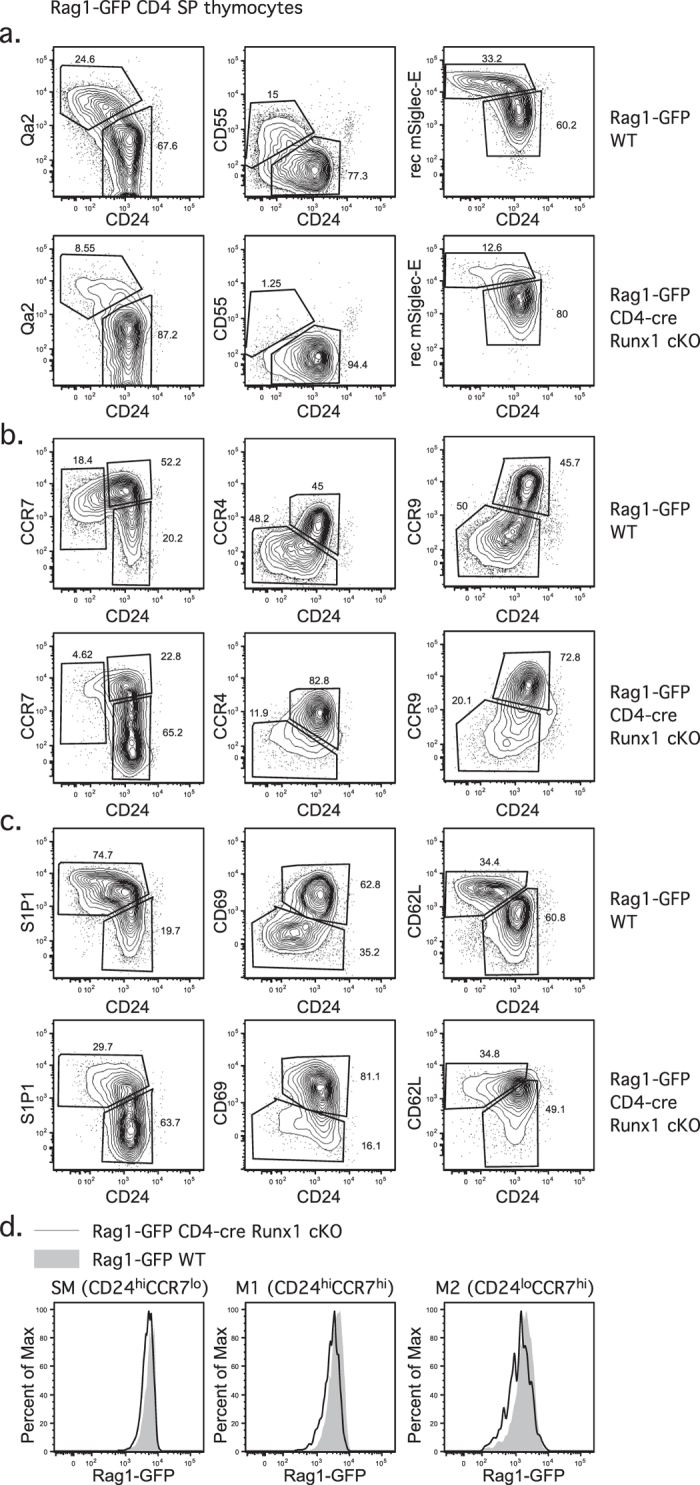
CD4 SP thymocytes from CD4-cre Runx1 cKO mice have a block in intra-thymic maturation prior to thymic egress. (**a**) The expression of maturation markers Qa2, CD55 and mSiglec-E ligands relative to CD24 was examined in Rag1-GFP^+^ CD4 SP thymocytes from Rag1-GFP WT and Rag1-GFP CD4-cre Runx1 cKO mice. Data shown were representative FACS plots from five Rag1-GFP WT and five Rag1-GFP CD4-cre Runx1 cKO mice from five independent experiments. (**b**) The expression of chemokine receptors CCR7, CCR4 and CCR9 relative to CD24 was examined in Rag1-GFP^+^ CD4 SP thymocytes from Rag1-GFP WT and Rag1-GFP CD4-cre Runx1 cKO mice. Data shown were representative FACS plots from at least four Rag1-GFP WT and four Rag1-GFP CD4-cre Runx1 cKO mice from three independent experiments. (**c**) The expression of S1P1, CD69 and CD62L relative to CD24 was examined in Rag1-GFP^+^ CD4 SP thymocytes from Rag1-GFP WT and Rag1-GFP CD4-cre Runx1 cKO mice. Data shown were representative FACS plots from five Rag1-GFP WT and five Rag1-GFP CD4-cre Runx1 cKO mice from five independent experiments. (**d**) The Rag1-GFP intensity was examined in CD24^hi^CCR7^lo^ (SM) CD4 SP thymocytes, CD24^hi^CCR7^hi^ (M1) CD4 SP thymocytes and CD24^lo^CCR7^hi^ (M2) CD4 SP thymocytes from Rag1-GFP WT (grey histogram) and Rag1-GFP CD4-cre Runx1 cKO mice (solid line). Representative FACS analysis from at least six mice in each group from six independent experiments is shown.

SP thymocyte maturation can be delineated into different stages by various surface markers (reviewed in^3^). Our data indicates that CD4-cre Runx1 cKO mice have a block in T cell maturation. To determine when this block initiates (intra-thymic or post-thymic), we examined different markers that change with maturation (Qa2, CD55 and rec mSiglec-E). There is a decreased percentage of “mature” CD4 SP thymocytes (denoted as Qa2^hi^CD24^−^ or CD55^+^ CD24^−^ or rec mSiglec-E^hi^CD24^−^) in CD4-cre Runx1 cKO mice (Fig. 4a), confirming that there is an intra-thymic maturation defect in Runx1-deficient CD4 SP thymocytes. The expression of chemokine receptors involved in intra-thymic migration and thymic egress also changes with maturation^27^,^28^,^29^. Intra-thymic migration of T cells from cortex to medulla occurs concurrently with initiation of thymic maturation; hence, the expression of chemokine receptors, CCR7, CCR4 and CCR9, was examined in CD4 SP thymocytes from both WT and CD4-cre Runx1 cKO mice. As shown in Fig. 4b, using CCR7 and CD24, CD4 SP thymocytes can be divided into three populations: CD24^+^ CCR7^−^, semi-mature (SM); CD24^+^ CCR7^+^, mature 1 (M1); CD24^−^CCR7^+^, mature 2 (M2)^3^. The transition of CD4 SP thymocytes from SM to M1 to M2 is severely compromised in CD4-cre Runx1 cKO mice (Fig. 4b, left column). Similarly, a reduced percentage of CCR4^−^CD24^−^ or CCR9^−^CD24^−^ mature CD4 SP thymocytes was also found in CD4-cre Runx1 cKO mice. Thus, many changes associated with maturation fail to occur in CD4-cre Runx1 cKO mice, indicating a block at an early stage of thymic maturation.

Competency for thymic egress is gained at the M2 stage. The transcription factor KLF2 is expressed at the late M1 stage and promotes the expression of CD62L and S1P1^30^,^31^. Surface CD69 binds to and internalizes S1P1. When mature SP thymocytes downregulate CD69 expression, S1P1 is retained on the cell surface and senses S1P that is secreted by neural crest-derived pericytes around blood vessels^26^,^32^, initiating thymic egress. S1P1^+^ CD69^−^CD62L^+^ M2 SP thymocytes exit from the thymus into the blood and travel to T cell zone in the secondary lymphoid organs via CD62L and CCR7^33^,^34^. As shown in Fig. 4c, the percentage of S1P1^hi^CD69^−^ M2 population is decreased in CD4-cre Runx1 cKO mice. However, there is only a slightly reduced Rag1-GFP intensity of M2 CD4 SP thymocytes between WT and CD4-cre Runx1 cKO mice (Fig. 4d), indicating that thymic egress is only slightly delayed in Runx1-deficient M2 CD4 SP thymocytes. If egress was severely compromised in Runx1-deficient CD4 SP thymocytes, a substantial decrease in Rag1-GFP expression demonstrating thymic retention would have been observed.

### Defective sialylation in CD4^+^ T cells from CD4-cre Runx1 cKO mice

**Figure 5:**
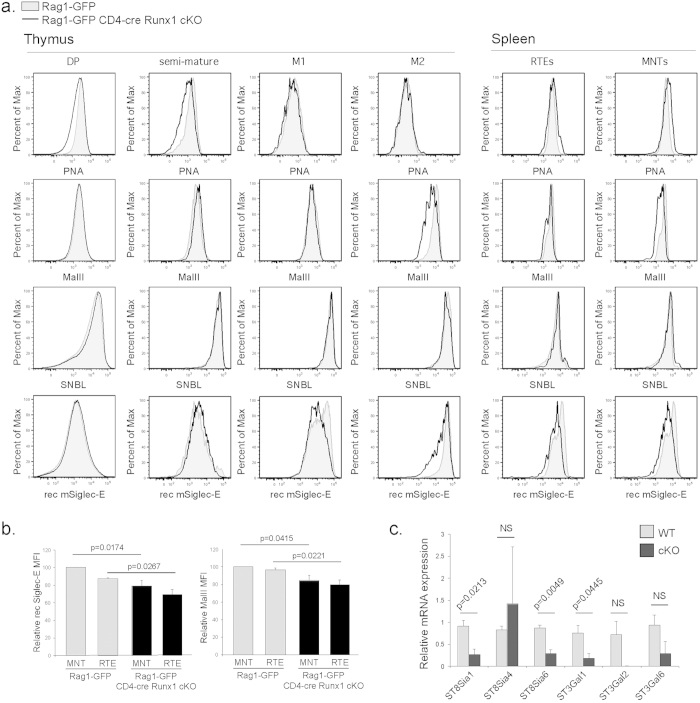
Defective sialylation in CD4^+^ T cells from CD4-cre Runx1 cKO mice. (**a**) DP (CD4^+^ CD8^+^), SM CD4 SP (CD4^+^ CD24^hi^CCR7^lo^), M1 CD4 SP (CD4^+^ CD24^hi^CCR7^hi^), M2 CD4 SP (CD4^+^ CD24^lo^CCR7^hi^) thymocytes, splenic CD4^+^ RTEs (CD4^+^ CD62L^+^ CD44^−^Rag1-GFP^+^), CD4^+^ MNTs (CD4^+^ CD62L^+^ CD44^−^Rag1-GFP^−^) from Rag1-GFP WT (grey histogram) and Rag1-GFP CD4-cre Runx1 cKO mice (solid line) were examined for sialylation by using plant lectins PNA, SNBL, MAL II and recombinant mSiglec-E Fc chimera (rec mSiglec-E). Thymocytes were first gated on expression of Rag1-GFP to exclude recirculating mature T cells. Representative FACS analysis from at least three mice in each group from three separate experiments is shown. PNA recognizes core-1-*O* glycans that lack terminal sialic acid; SNBL recognizes α2,6-linked sialic acids; MAL II recognizes α2,3-linked sialic acids; and rec mSiglec-E preferentially binds to α2,8-linked sialic acids. (**b**) Analysis of relative changes in rec Siglec-E and MalII MFI in CD4^+^ RTEs and MNTs from Rag1-GFP WT and Rag1-GFP CD4-cre NKAP cKO mice. Data shown is from 4 Rag1-GFP WT and 4 Rag1-GFP CD4-cre Runx1 cKO from 4 independent experiments. Within each experiment, the relative MFI of rec Siglec-E or MalII was normalized to the binding present in WT MNTs (=100). Error bars represent SEM, and statistical analysis of the differences in MFI was performed using unpaired Student’s *t* test using GraphPad Prism. (**c**) Quantitative PCR analysis comparing mRNA levels of α2,8-sialyltransferases (ST8Sia1, ST8Sia4 and ST8Sia6) and α2,3-sialyltransferases (ST3Gal1, ST3Gal2, ST3Gal6) in sorted splenic CD4^+^ RTEs from Rag1-GFP WT or Rag1-GFP CD4-cre Runx1 cKO mice is shown. The relative expression of these genes was normalized to 18S rRNA. Data shown is the mean ± SEM of three mice in each group, with all data normalized to one of the WT mice for each gene analyzed. Data were analyzed for significance by unpaired Student’s *t* test using GraphPad Prism.

We have previously shown that incorporation of sialic acid into cell surface glycans increases during T cell maturation. Thymocytes and RTEs with maturation blocks fail to appropriately upregulate sialylation^6^,^7^. Loss of sialylation, such as experimentally through neuraminidase, leads to binding of natural IgM and activation of complement^12^,^13^. Therefore, a detailed analysis of sialylation during thymic development and peripheral T cell maturation was performed in CD4-cre Runx1 cKO mice. Peanut agglutinin (PNA) recognizes terminal glycan residues that lack sialic acids. As shown in Fig. 5a, PNA binding decreased with thymic maturation in both WT and Runx1-deficient CD4 thymocytes, indicating that total levels of cell surface sialic acid were similar. However, using *Maackia amurensis* lectin II (MAL II), which specifically recognizes α2,3-sialic acid linkages, we found that Runx1-deficient mature CD4 SP thymocytes have less α2,3-sialylation as compared to WT cells starting at M2 CD4 SP thymocytes and continuing into peripheral RTEs and MNTs. No changes in α2,6-sialylation, as demonstrated by *Sambucus nigra* bark lectin (SNBL) binding, were observed. Recombinant (rec) mSiglec-E preferentially binds to α2,8-linked sialic acids, and less rec Siglec-E binding was observed as well in CD4-cre Runx1 cKO mice starting at the M1 stage of thymic CD4^+^ SP maturation and continuing into peripheral RTEs and MNTs. These data indicates that CD4 SP thymocytes have specific defects in sialylation in both α2,3- and α2,8-linkages in the absence of Runx1 in peripheral RTEs and MNTs, which can contribute to susceptibility for natural IgM binding and deposition of complement. The relative decrease in binding of MalII and rec Siglec-E to Runx1-deficient RTEs and MNTs is quantified in Fig. 5b. Consistent with the lack of a maturation defect in CD8^+^ T cells from CD4-cre Runx1 cKO mice, there are similar levels of α2,3- and α2,8-sialylation (as shown by MalII and rec Siglec-E binding, respectively) between CD8 SP thymocytes and peripheral CD8^+^ T cells from WT and CD4-cre Runx1 cKO mice (Supplemental Fig. 4).

Previously, we demonstrated that the decreased incorporation of α2,8-linked sialic acids into cell surface glycans in NKAP- and HDAC3-deficient RTEs was due to decreased expression of sialyltransferases, in particular ST8Sia6^6^,^7^. CD4^+^ RTEs from Rag1-GFP WT and Rag1-GFP CD4-cre Runx1 cKO mice were examined for expression of genes responsible for α2,3- or α2,8-sialylations (ST3Gal1, ST3Gal2, ST3Gal6; ST8Sia1, ST8Sia4, ST8Sia6, respectively). Among the sialyltransferases examined, mRNA expression of ST8Sia1, ST8Sia6 and ST3Gal1 were significantly decreased Runx1-deficient CD4^+^ RTEs in comparison of WT controls (Fig. 5c). Thus, decreased cell surface expression of α2,3- or α2,8-linked sialic acids in Runx1-deficient CD4^+^ T cells is due to lower levels of mRNA expression of sialyltransferases.

### The paucity of splenic CD4^+^ T cells in CD4-cre Runx1 cKO mice is not due to mislocalization to a different secondary lymphoid organ

**Figure 6:**
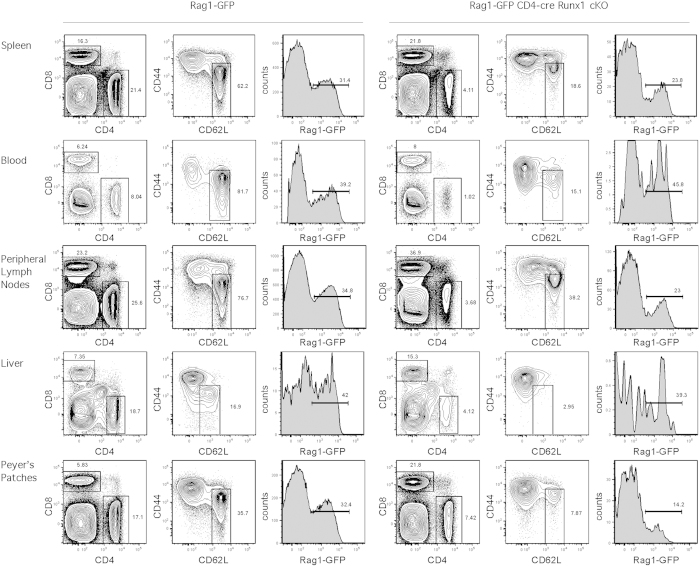
The paucity of splenic CD4^+^ RTEs in CD4-cre Runx1 cKO mice is not due to mislocalization. Peripheral lymphocytes isolated from spleen, blood, liver, peripheral lymph nodes (cervical, axillary, brachial and inguinal lymph nodes) and Peyer’s patches from Rag1-GFP WT or Rag1-GFP CD4-cre Runx1 cKO mice were examined for the proportion of CD4^+^ T, CD8^+^ T, naïve CD4^+^ T and CD4^+^ RTEs. FACS plots shown are of different organs obtained from a single Rag1-GFP WT or Rag1-GFP CD4-cre Runx1 cKO mouse in one experiment. Data shown are representative of four Rag1-GFP WT and four Rag1-GFP CD4-cre Runx1 cKO mice from four separate experiments.

Because we observed altered expression of CCR7 and CD62L in Runx1-deficient CD4 SP thymocytes, we considered whether the decreased number of peripheral CD4^+^ T cells in the spleen of CD4-cre Runx1 cKO mice resulted from altered homing to other secondary lymphoid organs (Fig. 6). However, no accumulation of CD4^+^ RTEs in any particular organ was found in CD4-cre Runx1 cKO mice, suggesting that the decreased number of Runx1-deficient CD4^+^ T cells is not due to the altered homing. The percentage of CD4^+^ T cells in CD4-cre Runx1 cKO mice is severely decreased in blood, peripheral lymph nodes, liver and Peyer’s patches as compared to WT mice. In particular, no naïve CD4^+^ T cells were present in the liver from CD4-cre Runx1 cKO mice. The complete loss of naïve CD4^+^ T cells in the liver is consistent with their elimination by complement, as the liver is the primary site of complement production^35^. Thus, decreased CD4^+^ T cells counts are globally observed in the periphery in CD4-cre Runx1 cKO mice.

### Positive and negative selection are not altered in CD4 SP thymocytes from CD4-cre Runx1 cKO mice

**Figure 7:**
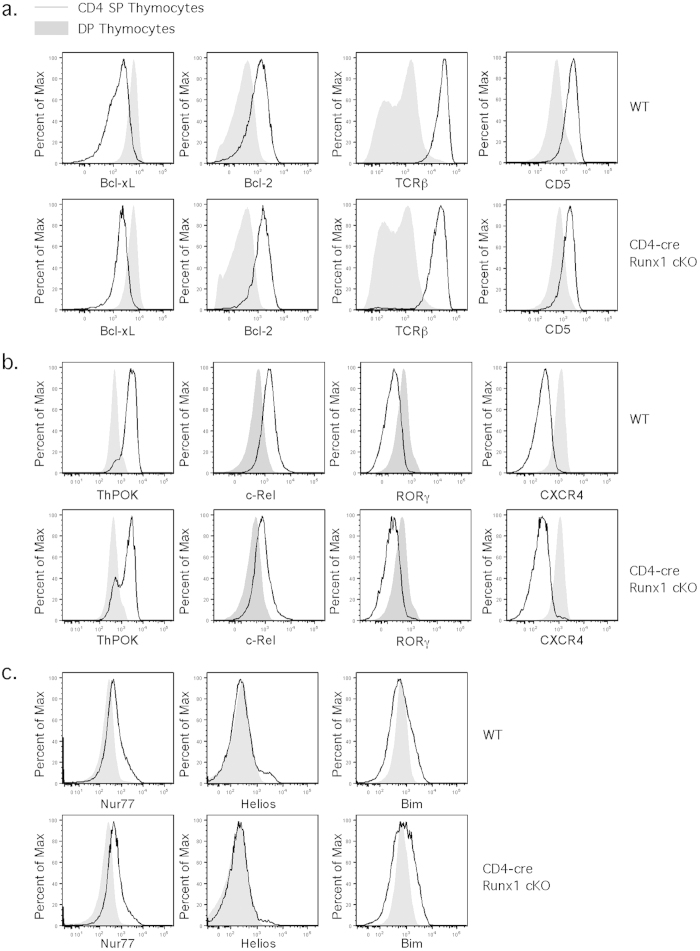
CD4-cre Runx1 cKO mice display normal positive selection and negative selection. (**a**) DP and CD4 SP thymocytes from Rag1-GFP WT and Rag1-GFP CD4-cre Runx1 cKO mice were examined for surface expression of TCRβ, CD5 and intracellular expression of Bcl-xL, Bcl-2. (**b**) DP and CD4 SP thymocytes from Rag1-GFP WT and Rag1-GFP CD4-cre Runx1 cKO mice were examined for intranuclear expression of ThPOK, c-Rel, RORγ, and surface expression of CXCR4. (**c**) DP and CD4 SP thymocytes from Rag1-GFP WT and Rag1-GFP CD4-cre Runx1 cKO mice were examined for intranuclear expression of Nur77, Helios, and intracellular expression of Bim. For surface and intracellular FACS, thymocytes were first gated on Rag1-GFP^+^ to exclude recirculating mature T cells from the analysis. For intranuclear FACS, as fluorescence of Rag1-GFP protein is disrupted by the use of Foxp3/Transcription factor buffer, thymocytes were not gated for Rag1-GFP expression. Grey histogram represents DP thymocytes while solid line represents CD4 SP thymocytes. Data shown are representative of at least four Rag1-GFP WT and five Rag1-GFP CD4-cre Runx1 cKO mice from four independent experiments.

To confirm that the thymic defect in the CD4 SP pool in CD4-cre Runx1 cKO mice is due to an early block in T cell maturation, we performed a thorough analysis of positive and negative selection. After positive selection, signaling through the TCR triggers upregulation of surface TCRβ and CD5^36^, and the induction of the anti-apoptotic molecule Bcl-2^37^. Another anti-apoptotic family member Bcl-xL is highly expressed in DP thymocytes and downregulated after positive selection^38^. We examined whether alterations in positive selection contributed to the changes observed in CD4 SP thymocytes in CD4-cre Runx1 cKO mice. However, the upregulation of TCRβ, CD5, and Bcl-2, and the downregulation of Bcl-xl, in CD4 SP thymocytes as compared to DP thymocytes were similar between WT and CD4-cre Runx1 cKO mice (Fig. 7a). The transcription factor RORγt is required for Bcl-xL expression that regulates the survival of DP thymocytes^38^, and downregulation of RORγt with positive selection is also important for the induction of c-Rel^39^. The transcription factor ThPOK is required for CD4 lineage commitment^40^,^41^. CXCR4 expression is also downregulated upon positive selection. As shown in Fig. 7b, the expression of ThPOK, RORγ, c-Rel and CXCR4 are all modulated normally in Runx1-deficient CD4 SP thymocytes after positive selection as well. Therefore, the defect in CD4 SP thymocytes from CD4-cre Runx1 cKO mice is not due to an alteration in positive selection or lineage commitment.

It is also possible that decreased percentage and number of CD4 SP thymocytes in CD4-cre Runx1 cKO mice could be due to enhanced negative selection. Mice overexpressing proapoptotic molecules such as Nur77 and Bim have increased negative selection, leading to decreased numbers of SP thymocytes^42^,^43^,^44^. In addition, the expression level of Helios is also an indicator of enhanced negative selection^4^. Thus, we examined whether there is an increased expression of Nur77, Helios and Bim in CD4 SP thymocytes in CD4-cre Runx1 cKO mice. As shown in Fig. 7c, the expression levels of Nur77, Helios and Bim in DP and CD4 SP thymocytes are comparable between WT and CD4-cre Runx1 cKO mice. This is consistent with previous work demonstrated that there was no increased negative selection in thymocytes from D011.10 TCR tg/CD4-cre Runx1 cKO mice that were stimulated with various concentration of OVA peptides^15^. Thus, the defect in CD4 SP thymocytes in CD4-cre Runx1 cKO mice is due to intra-thymic maturation and not defects in positive selection, negative selection or CD4 lineage commitment.

### Cell-intrinsic requirement of Runx1 for CD4^+^ T cell maturation

**Figure 8:**
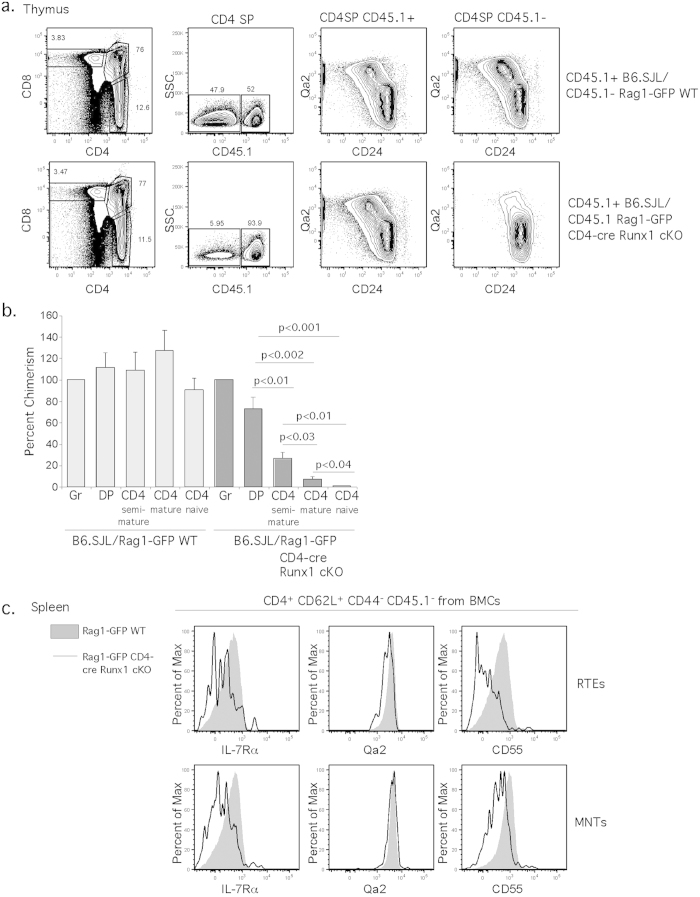
The defect in T cell maturation in the absence of Runx1 is cell intrinsic. Mixed bone marrow chimeras were generated using 1:1 mixture of bone marrow cells from either Rag1-GFP WT (CD45.2^+/+^)/B6.SJL (CD45.1^+/+^) or Rag1-GFP CD4-cre Runx1 cKO (CD45.2^+/+^)/B6.SJL (CD45.1^+/+^) into lethally irradiated congenic B6.SJL (CD45.1^+/+^) recipients. T cell development was analyzed 10 weeks after transplantation. DP thymocytes are CD4^+^ CD8^+^; semi-mature CD4 SP thymocytes are CD4^+^ CD8^−^CD24^+^ Qa2^−^; mature CD4 SP thymocytes are CD4^+^ CD8^−^CD24^−^Qa2^+^; splenic naïve CD4^+^ T cells are CD4^+^ CD8^−^CD62L^+^ CD44^−^. (**a**) CD4 SP thymocytes from Rag1-GFP WT (CD45.2^+/+^)/B6.SJL (CD45.1^+/+^) or Rag1-GFP CD4-cre Runx1 cKO (CD45.2^+/+^)/B6.SJL (CD45.1^+/+^) chimeras were analyzed by using CD45.1 and thymic maturation by using CD24 and Qa2. (**b**) The relative chimerism as compared to the granulocyte population was calculated. Error bars indicate SEM, and significances are determined by an unpaired Student’s *t* test. Data shown are the average of four Rag1-GFP WT (CD45.2^+/+^)/B6.SJL (CD45.1^+/+^) chimeras and four Rag1-GFP CD4-cre Runx1 cKO (CD45.2^+/+^)/B6.SJL (CD45.1^+/+^) chimeras from four independent experiments. (**c**) Splenic CD4^+^ CD62L^+^ CD44^−^CD45.1^−^Rag1-GFP^+^ RTEs and CD4^+^ CD62L^+^ CD44^−^CD45.1^−^Rag1-GFP^−^ MNTs from bone marrow chimeras were examined for the expression of IL-7Rα, Qa2 and CD55. Grey histogram represents cells from CD45.1^−^Rag1-GFP WT cells while solid line represents cells from CD45.1^−^Rag1-GFP CD4-cre Runx1 cKO cells from each bone marrow chimera.

To determine whether the CD4^+^ T cell maturation block in CD4-cre Runx1 cKO is cell-intrinsic, we analyzed mixed bone marrow chimeric (BMC) mice. The mice were reconstituted with an equal mixture of CD45.2^+^ Rag1-GFP WT or CD45.2^+^ Rag1-GFP CD4-cre Runx1 cKO with CD45.1^+^ B6.SJL total bone marrow cells and were analyzed after 10 weeks. Few mature CD4 SP thymocytes (CD24^lo^Qa2^hi^) derived from Rag1-GFP CD4-cre Runx1 cKO mice were observed, as compared to WT thymocytes from the same chimera, or the control (Rag1-GFP/WT) chimera (Fig. 8a). While the relative chimerism across all populations in mice reconstituted with Rag1-GFP WT bone marrow remains similar, however, relative chimerism of the Rag1-GFP CD4-cre Runx1 cKO mice decreased dramatically concurrently with T cell maturation (quantified in Fig. 8b). In the periphery (Fig. 8c), CD4^+^ RTEs and MNTs T cells derived from Rag1-GFP CD4-cre Runx1 cKO bone marrow exhibit decreased IL-7Rα and CD55 expression as well. Thus, the use of mixed BMC confirms that the defect in T cell maturation in CD4-cre Runx1 cKO is cell-intrinsic, and that Runx1 is essential for maturation of CD4^+^ T cells.

## Discussion

Although lack of peripheral CD4^+^ T cells in CD4-cre Runx1 cKO mice has been previously described^15^,^16^,^45^, the mechanism responsible for this observation was not understood. Decreased expression of IL-7Rα was thought to contribute to a defect in T cell homeostasis in CD4-cre Runx1 cKO mice. In this report, we identified the lack of peripheral CD4^+^ T cells in CD4-cre Runx1 cKO mice is not due to decreased IL-7Rα expression, but is due to complement-mediated depletion resulted from a failure in T cell maturation. Runx1 is not required for positive selection or negative selection. The transition from SM to M1 in CD4 SP thymocytes is severely compromised in CD4-cre Runx1 cKO mice, and consequently, resulting functional competencies such as TNFα licensing and protection from complement in the periphery are defective. Runx1-deficient peripheral naïve CD4^+^ T cells fail to produce TNF-α in response to TCR stimulation. Defective sialylation and IgM/complement protein deposition were found on the cell surface of Runx1-deficient CD4 T cells, leading to elimination of Runx1-deficient naïve CD4^+^ T cells in the periphery.

Previously, the Satake group generated Bcl-2 tg/CD4-cre Runx1 cKO mice^45^. Bcl-2 is a downstream target of IL-7R signaling. Consequently, if the peripheral naïve CD4^+^ T cell defect in CD4-cre Runx1 cKO mice is due to the IL-7-Bcl-2-axis for T cell survival and homeostasis, there should be a phenotypic and cellular rescue of naïve CD4^+^ population in Bcl-2 tg/CD4-cre Runx1 cKO mice or IL-7Rα/tg CD4-cre Runx1 cKO. However, similar to what we observed in IL-7Rα/tg CD4-cre Runx1 cKO mice, the percentage and number of naïve CD4^+^ T cells in Bcl-2 tg/CD4-cre Runx1 cKO mice remained low^45^, indicating that there is a different mechanism responsible for the paucity of peripheral naïve CD4^+^ T cells in CD4-cre Runx1 cKO mice. We identified complement-mediated elimination of Runx1-deficient CD4^+^ T cells as the major cause. Furthermore, when we examined blood CD4^+^ RTEs and MNTs from CD4-cre Runx1 cKO mice to identify when the complement-mediated elimination initiates, we found a substantial increase of IgM and complement deposition on blood CD4^+^ RTEs and MNTs as compared to splenic CD4^+^ RTEs and MNTs. We also found substantially increased IgM and complement deposition on RTEs from blood from Rag1-GFP WT mice. These data support the idea that immature T cells upon egress from the thymus the via cortico-medullary junctions are immediately tested for maturation by complement in the circulation. The thymus is a complement-privileged site that protects immature thymocytes from complement-mediated killing before they complete intra-thymic maturation^7^,^46^.

Unlike CD4-cre NKAP cKO and CD4-cre HDAC3 cKO mice that have normal SP thymocytes numbers and a defect in post-thymic maturation^6^,^7^, CD4-cre Runx1 cKO mice have an earlier block in thymic T cell maturation at the M1 SP stage. We have previously demonstrated that increased sialylation occurs concurrently with T cell maturation. The exposure of terminal GluNAc/Gal of *O*-glycan or *N*-glycan on DP thymocytes is lost by sialylation once thymocytes pass positive selection. Specifically, α2,3-sialic acid linkages and α2,8-sialic acid linkages increase during SP thymocyte cell maturation from semi-mature to mature stage^7^. Runx1-deficient CD4 SP thymocytes have less MAL II and rec mSiglec-E binding, indicating decreased α2,3- and α2,8-linked sialic acids, respectively, as compared to WT cells. In contrast, NKAP-deficient and HDAC3-deficient T cells primarily have a defect in α2,8-linked sialic acids. As incorporation of sialic acids onto surface glycoprotein is a crucial step during T cell maturation, the defect in both α2,3 and α2,8-linkage sialylation may explain why there is exacerbated natural IgM recognition and complement-mediated elimination in Runx1-deficient CD4^+^ T cells, leading to a decreased RTEs and MNTs in the periphery.

As Runx1-deficient CD4 SP thymocytes display a severe block in T cell maturation and have multiple alterations in cell surface proteins, including CCR7. Interestingly, CCR7 itself is not required for T cell maturation, as the Anderson group showed that CCR7/CCR4 double KO (dKO) mice have no defect in T cell maturation^29^. Indeed, both CCR7KO and CCR7/CCR4 dKO mice have similar number and percentage of mature CD4 SP (CD69^−^Qa2^+^) thymocytes as compared to WT mice. Therefore, decreased CCR7 expression is not the cause the defect in intra-thymic maturation in CD4-cre Runx1 cKO mice. Qa2, a member of nonclassical MHC class Ib molecule, is extensively used as a standard marker for T cell maturation in the thymus and periphery^47^. However, a functional role of Qa2 during T cell maturation has not been demonstrated. We previously showed that HDAC3-deficient CD4^+^ T cells have expression of Qa2 similar to WT cells, although HDAC3-deficient naïve T cells are both functionally immature and have decreased expression of other markers associated with T cell maturation such as CD55 and α2,8 sialylation. Here, we show that CD4^+^ RTEs and MNTs from CD4-cre Runx1 cKO mice have normal expression of Qa2, but are similarly defective in maturation. Thus, our data from both CD4-cre Runx1 cKO mice and CD4-cre HDAC3 cKO mice demonstrate that normal Qa2 expression in the periphery cannot be used as a marker to indicate that maturation has occurred in the periphery. Rather, it is necessary to examine functional changes such as TNFα licensing, in addition to changes in the expression of multiple proteins on the cell surface, to determine whether T cell maturation has occurred, rather then relying solely on Qa2.

## Methods

### Mouse

Floxed Runx1 mice were generated by Dr. Nancy Speck (University of Pennsylvania, Philadelphia, PA) and purchased from The Jackson Laboratory (B6.129-Runx1^tm3.1Spe^/J). Rag1-GFP knock-in mice were kindly provided by Dr. Nobuo Sakaguchi (Kumamoto University, Kumamoto, Japan)^23^. IL-7Rα transgenic mice were previously described and provided by Dr. Al Singer (National Cancer Institute, Bethesda, MD)^48^. C57BL/6 (B6) mice were purchased from The Jackson Laboratory, and the congenic B6.SJL mice were purchased from National Cancer Institute at Frederick. All mice were housed in a specific pathogen-free barrier facility at Mayo Clinic, and were analyzed at 8–12 weeks of age. All animal experiments were conducted with approval from the Institutional Animal Care and Use Committee at Mayo Clinic, following NIH guidelines. “WT” littermates used as controls in each experiment included Runx1 floxed/cre^−^, Runx1 WT/cre^+^, or Runx1 WT/cre^−^ mice, as T cell maturation in these mice is indistinguishable from WT B6 mice. Littermates were used whenever possible as controls, and otherwise age-matched controls were used.

### Flow cytometry

Spleens and thymi from indicated mice were obtained and processed as single-cell suspensions before FACS analysis. The following antibodies were purchased from BD Bioscience, BioLegend, eBioscience, R&D Systems, Tonbo Bioscience, Cell Signaling Technology or Cedarlane: Bcl-2 (BCL/10C4), Bcl-xL (7B2.5), Bim (H-191), CCR4 (2G12), CCR7 (4B12), CCR9 (9B1), CD4 (RM4-5, GK1.5), CD45.1 (A20), CD5 (53-7.3), CD8α (53-6.7), CD24 (M1/69), CD44 (IM7), CD45RB (C363-16A), CD55 (RIKO-3), CD62L (MEL-14), CD69 (H1.2F3), complement C1q (RMC7H8), complement C3 (RMC11H9), complement C4 (RMC16D2), CXCR4 (2B11), Helios (22F6), IgM (RMM-1), IL-7Rα (A7R34), Nur77 (12.14), c-Rel (1RELAH5), RORγ (B2D), recombinant mSiglec-E-Fc chimera, Runx1 (RXDMC), S1P1 (713412), TCRβ (H57-597), ThPOK (2POK), TNF-α (MP6-XT22) and Qa2 (695H1-9-9). Biotinylated plant lectins (MAL II, PNA and SNBL) were purchased from Vector Laboratories. For intracellular FACS, cells were stained with fixable viability dye (FVD; eBioscience and Tonbo) before surface stain. Following the surface stain, cells were fixed and permeablized with IC Fixation Buffer (eBioscience) and stained for Bcl-2, Bcl-xL, or Bim. For intranuclear FACS, cells were stained with FVD before surface stain. After the surface stain, cells were fixed and permeablized with Foxp3/Transcription Factor Staining Buffer Set (eBioscience) and stained for Nur77, c-Rel, RORγ, Runx1 or ThPOK. All data were doublet excluded with forward light scatter width/height and side light scatter width/height, and dead cells were excluded with FVD before analysis. Samples were acquired using LSRII (BD Bioscience) or Attune NxT (Life Technologies) flow cytometers and analyzed with FlowJo software (FlowJo, LLC.)

### Blood cell preparation and complement deposition assays

Whole blood was isolated from indicated mice and mixed with 20μM/ml heparin at a 50:50 ratio. The red blood cells were lysed by cold ACK buffer for 10 minutes on ice and then were washed twice with cold 1×PBS before complement deposition assays. For complement experiments, ACK buffer-treated blood cells or splenocytes were incubated in GVB^++^ buffer (Complement Technology) for 1 hour at room temperature before staining with indicated Abs.

### Cell stimulation and intracellular cytokine assays

Total splenocytes were cultured for 16 hours at 37 °C in 5% CO_2_ in complete medium containing RPMI 1640 (Life Technologies), 10% fetal calf serum, 100 U/ml penicillin-streptomycin (Life Technologies), and 2 mM L-glutamine (Life Technologies), and were stimulated accordingly. For TNF-α intracellular stain, 5 × 10^6^ splenocytes were stimulated with or without plate-coated α-CD3ε (10μg/ml, 145-2C11; Bio-X-Cell) plus soluble α-CD28 (1μg/ml, 37.51; Bio-X-Cell) in the presence of Protein Transport Inhibitor Cocktail (eBioscience). The following day, cells were harvested and washed once with 1× PBS and stained with FVD before surface stain. Cells were then fixed and permeablizied with IC Fixation Buffer (eBioscience) and stained for intracellular TNF-α (eBioscience).

### FACS sorting and real-time Q-PCR analysis of relative mRNA expression

To isolate splenic CD4^+^ RTEs, cells from Rag1-GFP WT and Rag1-GFP CD4-cre Runx1 cKO mice were harvested and first subject to negative selection using a biotin-labeled cocktail of antibodies (B220, CD11b, Ter119, Gr-1, CD19, CD11c and CD8) to eliminate unwanted populations using magnetic bead selection (Miltenyi Biotec) prior to sorting. All sorting was done on a FACSAria (BD Bioscience), and RTEs were sorted by gating on CD4^+^ CD44^−^CD62L^+^ Rag1-GFP^+^. mRNA was later isolated from sorted CD4^+^ RTEs using an RNeasy Mini kit (QIAGEN). cDNA was then generated and amplified with the Ovation PicoSL WTA V2 kit (NuGen). The mRNA expression was measured using TaqMan probes (Applied Biosystems) for ST8Sia1, ST8Sia4, ST8Sia6, ST3Gal1, ST3Gal2 and ST3Gal6. TaqMan probe for 18S rRNA is used as the internal control. Samples were analyzed using an ABI RT-PCR StepOne Plus System (Applied Biosystems), and relative gene expression was calculated via the 2-ΔΔCT method. Data shown is from independent isolations of CD4+ RTEs from 3 Rag1-GFP WT and 3 Rag1-GFP CD4-cre Runx1 cKO mice.

### Generation of mixed bone marrow chimera

Recipient B6.SJL mice were given 1000 rad in one dose and rested for at least 5 hours before receiving mixed bone marrow cells. Mixed BMCs were generated by i.v. injection of 4 × 10^6^ whole bone marrow cells from either mixes of Rag1-GFP WT (CD45.2^+/+^)/B6.SJL (CD45.1^+/+^) or Rag1-GFP CD4-cre Runx1 cKO (CD45.2^+/+^)/B6.SJL (CD45.1^+/+^) at a 1:1 ratio into irradiated congenic B6.SJL (CD45.1^+/+^) recipients. Chimeric mice received enrofloxacin in their drinking water for 3 weeks and were analyzed after 10 weeks.

### Statistical Analysis

Unpaired student’s *t* test was used to calculate statistical significance between groups and the calculation was performed with GraphPad Prism software. p < 0.05 was considered statistical significant. Data are shown as mean ± SEM.

## Additional Information

**How to cite this article**: Hsu, F.-C. *et al.* An Essential Role for the Transcription Factor Runx1 in T Cell Maturation. *Sci. Rep.* **6**, 23533; doi: 10.1038/srep23533 (2016).

## Supplementary Material

Supplementary Information
