# Supplementary material for: An Essential Role for the Transcription Factor Runx1 in T Cell Maturation
Source: Sci Rep. 2016 Mar 29;6:23533. doi: 10.1038/srep23533 (PMC4810436; doi:10.1038/srep23533)
Supplement: Supplementary Information [file srep23533-s1.pdf]

An Essential Role of Transcription Factor Runx1 for T Cell Maturation

Fan-Chi Hsu[1], Michael J. Shapiro[1], Barsha Dash[1], Chien-Chang Chen[1], Megan M. Constans[1], Ji Young Chung[1], Sinibaldo R. Romero Arocha[1], Paul J. Belmonte[1], Meibo W. Chen[1], Douglas C. McWilliams[1], and Virginia Smith Shapiro[1]

[1]Department of Immunology, Mayo Clinic, Rochester, MN, United States

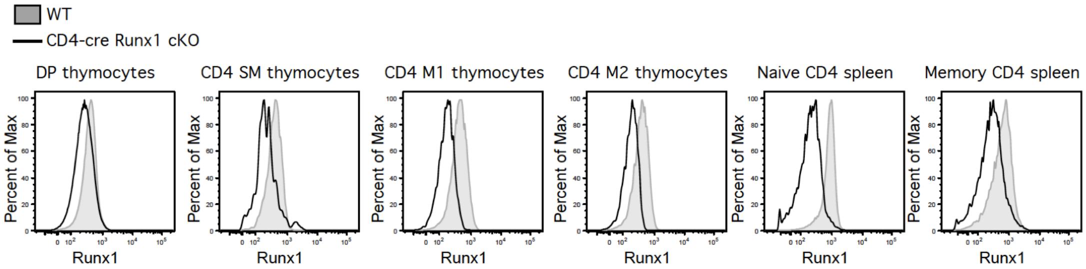

Supplemental Figure 1

Runx1 protein expression throughout T cell development in CD4-cre Runx1 cKO mice and WT mice

Intranuclear Runx1 staining was performed on thymocytes and splenocytes from WT and CD4-cre Runx1 cKO mice. The protein levels of Runx1 in DP ($CD4^{+}CD8^{+}$), CD4 SM ($CD4^{+}CD8^{-}CD24^{hi}CCR7^{lo}$), CD4 M1 SP ($CD4^{+}CD8^{-}CD24^{hi}CCR7^{hi}$) thymocytes, CD4 M2 SP ($CD4^{+}CD8^{-}CD24^{lo}$CCR7hi) thymocytes, naïve ($CD4^{+}CD62L^{+}CD44^{-}$) and memory ($CD4^{+}CD62L^{-}CD44^{+}$) T cells from both WT and CD4-cre Runx1 cKO mice were examined. As intranuclear staining destroys GFP fluorescence, expression of Runx1 in RTEs was not examined. Data shown are representative of five WT and five Rag1-GFP CD4-cre Runx1 cKO mice from five separate experiments.

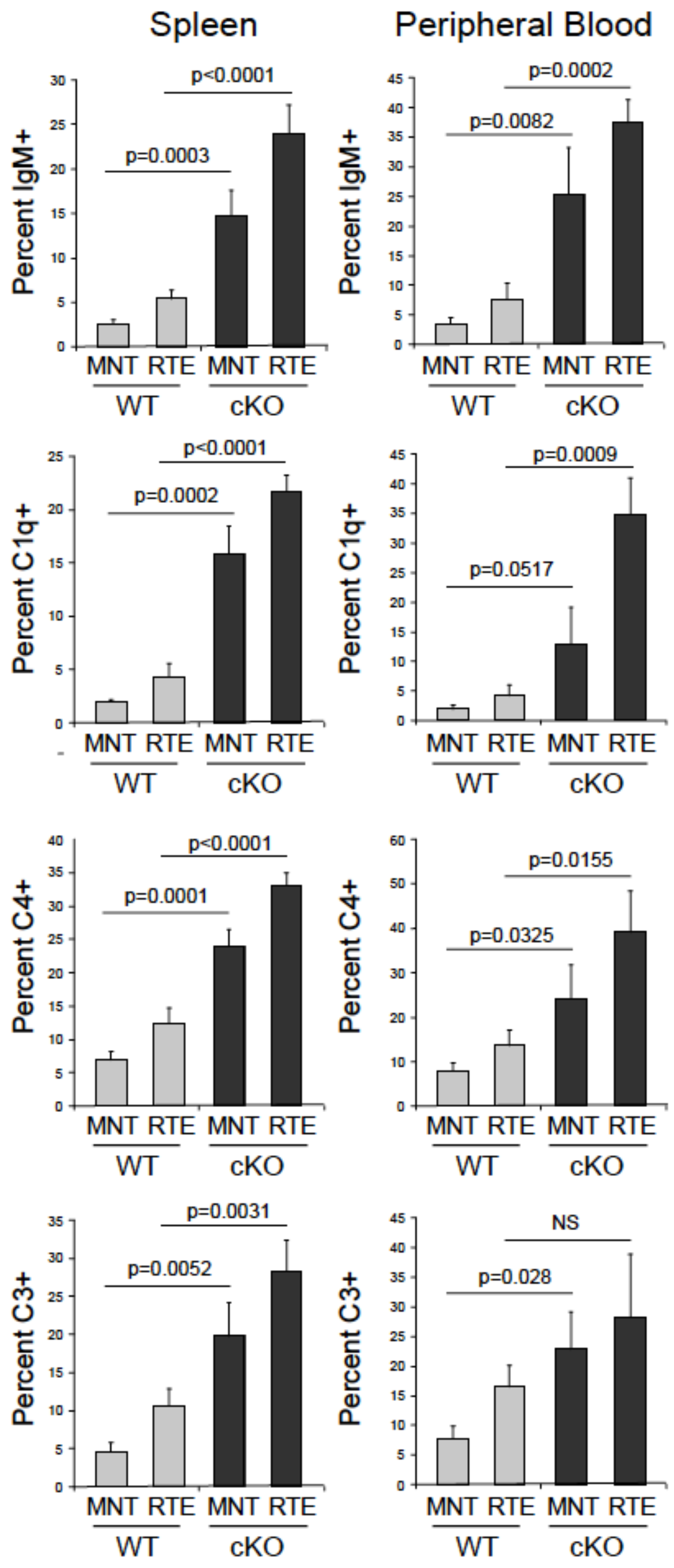

Spleen
Peripheral Blood
Percent IgM+
p<0.0001
p=0.0003
p=0.0002
p=0.0082
Percent C1q+
p<0.0001
p=0.0002
p=0.0009
p=0.0517
Percent C4+
p<0.0001
p=0.0001
p=0.0155
p=0.0325
Percent C3+
p=0.0031
p=0.0052
NS
p=0.028
MNT RTE MNT RTE
WT cKO

Supplemental Figure 2

Quantitation of IgM and complement protein deposition on splenic and peripheral blood Runx1-deficient $CD4^+$ RTEs and MNTs as compared to WT

Deposition of IgM, C1q, C4 and C3 in splenic (left column) and peripheral blood (right column) $CD4^+$ RTEs and MNTs from Rag1-GFP WT and Rag1-GFP CD4-cre Runx1 cKO mice as shown in Figure 3 was quantified across several experiments. For analysis of complement deposition in splenic $CD4^+$ T cells, the results shown are quantified from at least 6 Rag-GFP WT and 5 Rag-GFP CD4-cre Runx1 cKO mice from 5 independent experiments. For the analysis of complement deposition in peripheral blood CD4+ T cells, the results shown are quantified from at least 6 Rag1-GFP WT and 3 Rag1-GFP CD4-cre Runx1 cKO mice. Error bars represent SEM, and significance was determined by unpaired Student's *t* test using GraphPad Prism.

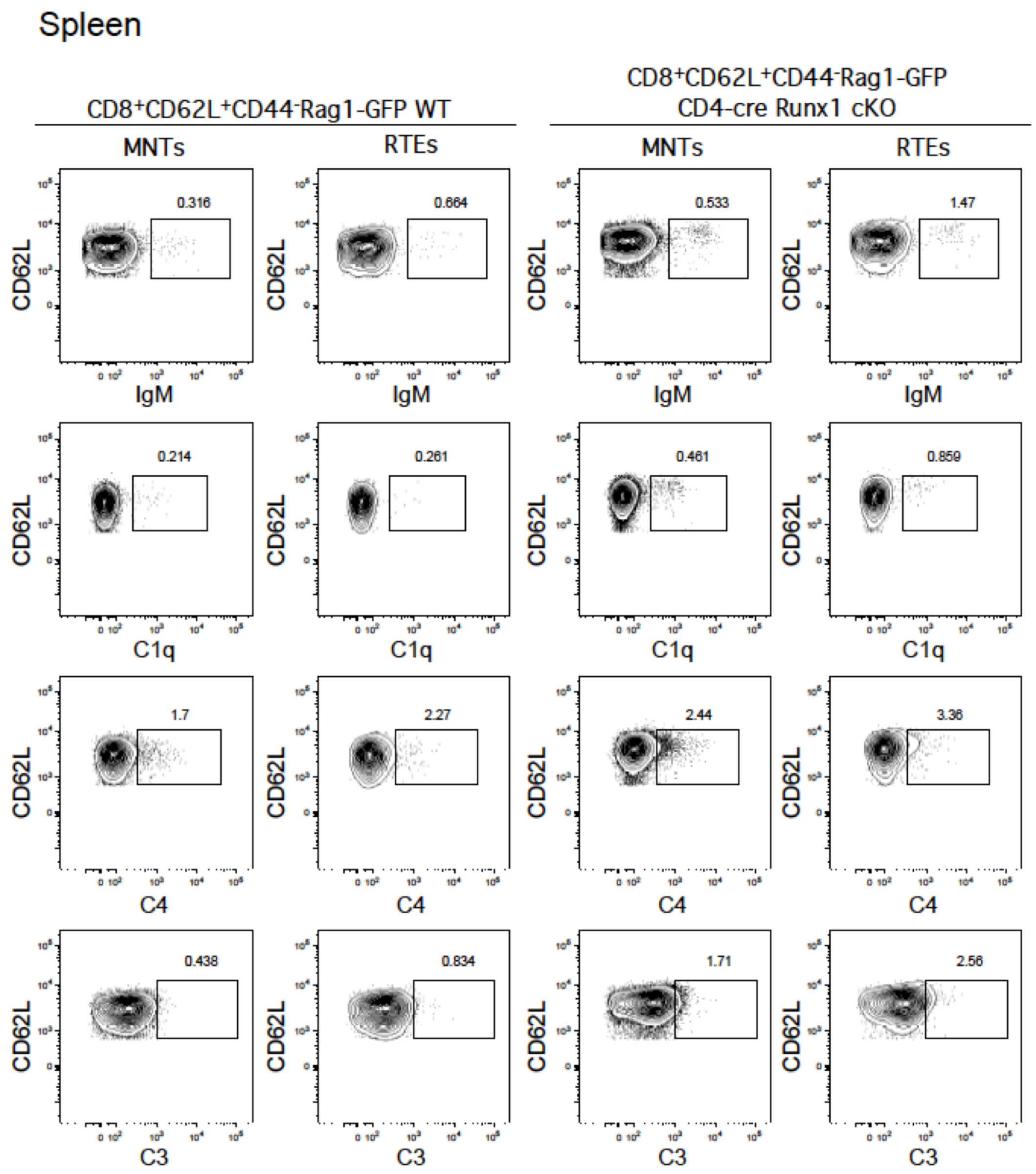

Supplemental Figure 3

IgM, C1q, C4 and C3 are not deposited on CD8$^+$ T cells from CD4-cre Runx1 cKO mice

Splenic CD8$^+$ RTEs and MNTs form Rag1-GFP WT and Rag1-GFP CD4-cre Runx1 cKO mice were examined for the deposition of IgM, C1q, C4 and C3 as denoted in the figure. Cells were incubated in GVB^{++} buffer prior to staining with indicated Abs. CD8$^+$ RTEs were

gated on CD62L$^+$CD44$^-$Rag1-GFP$^+$ while CD8$^+$ MNTs were gated on CD62L$^+$CD44$^-$Rag1-GFP$^-$. Data shown are representative of six Rag1-GFP WT and six Rag1-GFP CD4-cre Runx1 cKO mice from five separate experiments.

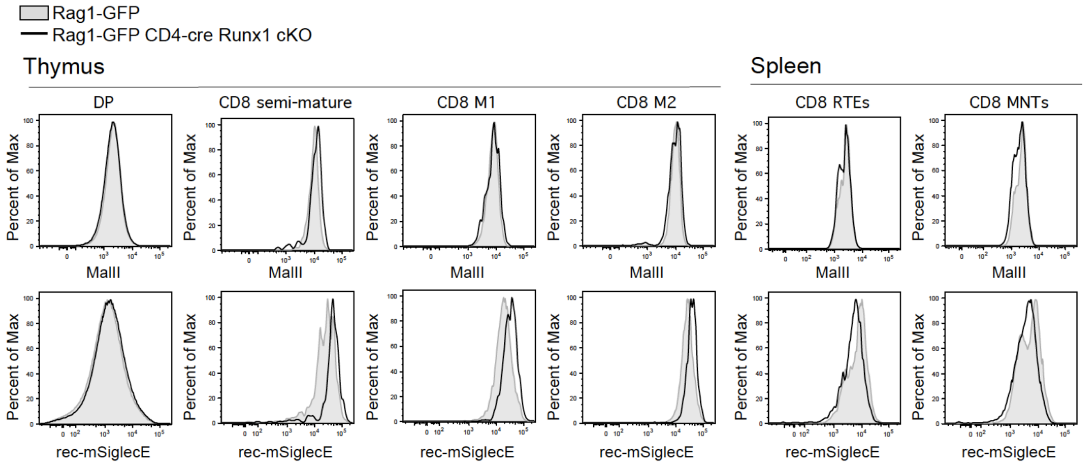

Supplemental Figure 4

α2,3- and α2,8-sialylation on CD8 SP thymocytes and $CD8^+$ T cells is not altered from CD4-cre Runx1 cKO mice

DP ($CD4^+CD8^+$), SM CD8 SP ($CD8^+CD24^{hi}CCR7^{lo}$), M1 CD8 SP ($CD8^+CD24^{hi}CCR7^{hi}$), M2 CD8 SP ($CD8^+CD24^{lo}CCR7^{hi}$) thymocytes, splenic $CD8^+$ RTEs ($CD8^+CD62L^+CD44^-$Rag1-GFP^+), $CD8^+$ MNTs ($CD8^+CD62L^+CD44^-$Rag1-GFP^-) from Rag1-GFP WT (grey histogram) and Rag1-GFP CD4-cre Runx1 cKO mice (solid line) were examined for α2,8-sialylation by using recombinant mSiglec-E Fc chimera (rec mSiglec-E) and α2,3-sialylation using MalII. Thymocytes were first gated on expression of Rag1-GFP to exclude recirculating mature T cells. Representative FACS analysis from at least three mice in each group from three separate experiments is shown.
